# Supplementary material for: Evolutionary Study of the Crassphage Virus at Gene Level
Source: Viruses. 2020 Sep 17;12(9):1035. doi: 10.3390/v12091035 (PMC7551546; doi:10.3390/v12091035)
Supplement: Supplementary file 1 [file viruses-12-01035-s001.zip › viruses-909877 supplementary after minor rev/viruses 909877 supplementary.pdf]

viruses

Supplementary material

Evolutionary study of the crAssphage virus at gene level

Alessandro Rossi [1], Laura Treu [1,*] Stefano Toppo [2], Henrike Zschach [3], Stefano Campanaro [1,4] and Bas E. Dutilh [5]

[1] Department of Biology, University of Padova; alessandro.rossi.23@phd.unipd.it, laura.treu@unipd.it
[2] Department of Molecular Medicine, University of Padova; stefano.toppo@unipd.it
[3] Department of Biology, University of Copenhagen; henrike.zschach@bio.ku.dk
[4] CRIBI Biotechnology Center, University of Padua; stefano.campanaro@unipd.it
[5] Institute of Biodynamics and Biocomplexity, University of Utrecht; bedutilh@gmail.com
* Correspondence: laura.treu@unipd.it; Tel.: +39-049-8276165 (L.T.)

Index

Table S1 - The table includes the starting database of the project: the original IDs of the 805 crAss-like contigs, the IDs used in the study and the accession number of the project deposited at NCBI database.
Table S2 - The table includes the names of all homologous groups of proteins and the corresponding values for all statistics.
Table S3 - The table includes results of the 3D function prediction softwares Argot 2.5 and I-TASSER run on the Uncharacterized protein sequences, and the consensus of the two softwares.
Figure S1 -Distribution of the contig length before and after filtering step.
Figure S2 - Phylogenetic trees describing the most important gene-level evolutionary events found: two gene duplications and one intron insertion.
Figure S3 - Distribution of average Shannon information content of the homologous protein clusters, representing the conservation degree, across functional groups.
Figure S4 - Distribution of average Pearson coefficients across functional groups.

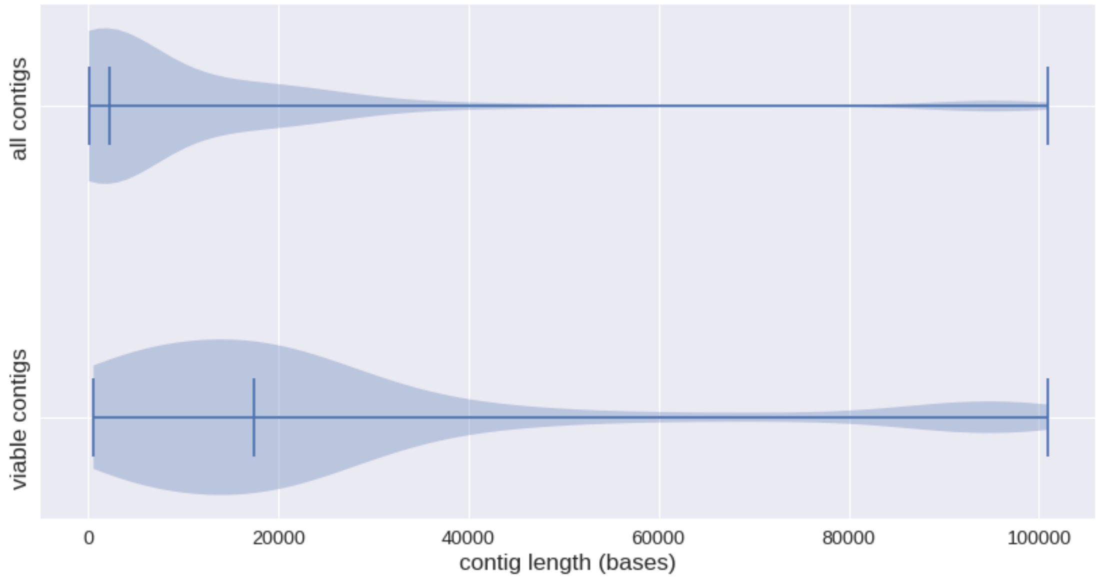

Figure S1. Length distribution of crAss-like contigs, showing the medians. The filtering step eliminated many of the short contigs, bringing the total number from 805 to 370 and raising the median length from 2,291 to 17,485 bases.

Duplication is one of the main phenomena that fuel gene-level evolution [1]. It is more common in dsDNA viruses compared to other kinds of viruses [2]. After the duplication, the two homologous sequences can undergo different evolutionary paths, splitting the functions of the original gene (i.e. subfunctionalization); likewise, one sequence may develop entirely new purposes (i.e. neofunctionalization) [3]. The duplication could also be exploited by the organism as a way to up-regulate the gene expression [4,5]. A frequent destiny, though, is for one of the two copies to accumulate mutations under neutral evolution that render it useless; for this reason, we speculate that the duplication of RepL has been beneficial to the virus, and has thus been favored by natural selection. This hypothesis is even more appealing when considering the high impact of purifying selection on viral evolution, due to their large population size [6,7]. The gene relative to the RepL protein was found in two copies in the crAssphage reference genome, named Reference_crAssphage.1_45 and Reference_crAssphage.1_91. The latter sequence (219 aa) is longer than the former (185 aa); the presence of two paralogs with similar lengths was verified in a total of 16 contigs. The paralogs arose through an ancient gene duplication, as supported by the RepL phylogenetic tree, which shows a distinct separation between the two ORFs (Figure S2a). This last observation is further corroborated by our finding of two copies of the gene in a contig coming from baboon gut metagenomic datasets (contig ID: Baboon.2.2). RepL proteins, which were first found in Staphylococcus aureus plasmids, are known to increase the number of copies of the plasmids they're found in; this prompts us to speculate that the duplication has led to a faster reproduction of the virus, and thus to the conservation of the duplicated sequence. Besides the aforementioned RepL genes, a total of three gene duplications has been newly detected in the collection of crAss-like phage contigs, including duplications of Reference_crAssphage.1_44, Reference_crAssphage.1_73, and Reference_crAssphage.1_74. Not much can be said about the other duplicated genes; most duplication events are found in single genomes, and it is then impossible to tell whether they're advantageous or not. One possible exception is Reference_crAssphage.1_74, which has been found duplicated in six related genomes; this gene, though, is one of the many that were not annotated and present top-performing methods as Argot2.5 and I-TASSER have not been able to unequivocally predict a putative function though a weak hypothesis has emerged and this protein could be a potential kinase. It is unlikely that duplicated genes are the product of an insertion as is the case with the Dut dUTPase, as the paralogs do not appear to align with different regions of the reference protein sequence. The first has been classified as a membrane protein and is located in the replication module; the duplication is found in a single contig coming from a Canadian sample. The paralogous genes are in close position in the genome and are both encoded on the forward strand. One of the two copies appears to be shorter and highly divergent from the other sequences in the homologous protein group, suggesting an accumulation of deleterious mutations. An additional duplication of the short paralog of the RepL protein is present in a single genome (Personal_sample.1, Table S1), which is thus the only one featuring three RepL copies. In particular, Reference_crAssphage.1_73 encodes for a tail sheath protein that, following the reference genome structure, is included in the short module composed of tail and structural proteins. Finally, six contigs contain duplications of the Reference_crAssphage.1_74 sequence (i.e. Gut.14, Gut.03, Gut.07, Gut.05, Activated_sludge.3 and Gut.06). In the phylogenetic tree of the protein these sequences are very closely related (Figure S2b). No annotation is available for this ORF; it can be hypothesized that it encodes for a structural protein, as it is surrounded, in the reference genome, by structural protein-encoding genes, and it is in proximity to capsid genes. This is confirmed by the iVIREONS structural protein score (0.62) and HHTOP prediction of transmembrane structure [9]. Argot2.5 failed to predict a function for this ORF, whereas I-TASSER predicted a kinase with a putative ATP-binding pocket with low confidence (Table S2). Furthermore, these six contigs containing the gene duplication (i.e. Gut.03, Gut.05, Gut.06, Gut.07, Activated_sludge.3, and Gut.14) are rather distant from the reference crAssphage genome and many of their ORFs do not match

with the reference. A final remark has to be made regarding the impact that incomplete contigs have in the investigation of gene duplications: a genome which has not been completely assembled and remains split in two or more contigs has, first and foremost, a chance of having missing genes; more importantly, paralogs may be located in different contigs representing fragments from the same genome, thus potentially preventing the recognition of duplicated genes.

Another protein cluster that contains two ORFs encoded on the crAssphage reference genome includes Reference_crAssphage.1_34 and Reference_crAssphage.1_36. According to the functional annotation, these ORFs are recognized as two distinct regions of a dUTPase gene that are split by the insertion of an intron-encoded endonuclease belonging to the HNH protein family, Reference_crAssphage.1_35. The intron insertion is relatively recent and was found in nine contigs, all of which were assembled from metagenomes of the twin sisters of a single family [8]. This can also be seen in the phylogenetic tree where the sequences having the insertion form a single clade (Figure S2c). There is no doubt about the recency of the HNH insertion. The protein cluster that includes this putative HNH endonuclease is among the smallest, featuring only nine sequences, and the position within the genome of the gene coding for the proteins belonging to this cluster is conserved, being always located between the two ORFs coding for the previously mentioned dUTPase. This is also shown by their close phylogenetic relationship clustering on the phylogenetic tree, and by the origin of the samples in which the insertion was identified; in fact they all originate from the metagenomic samples of the gut of two twin sisters. These assembled genomes are very closely related, as they cluster together among the other sequences coming from the Reyes study [9].

The intron is not found, nor the dut gene is split, in any other contig. The dUTPase protein family is widespread both among prokaryotes and eukaryotes, where it plays a role of great importance in avoiding deleterious mutations by preventing uracil to be integrated into the genome, and among retroviruses as well [10,11]. Given the mosaic nature of viral genomes, this means that the dut gene found in crAss-like viruses could have been incorporated early in the clade's evolution by means of HGT from a great number of other organisms. Since dUTPase proteins have evolved independently in many lineages of hosts and viruses, it would be interesting to investigate for the actual origin of this specific enzyme, whether dUTPases are present in Bacteroides sp. and whether such enzymes are related to the gene found in crAss-like family. The same could be speculated for the HNH endonuclease: it would be easy to assume that it originated from a similar protein found in a Bacteroides genome. The actual history of the single crAssphage strain remains uncertain, as well as the influence of the insertion in viral fitness. Still, it is sensible to hypothesize that this mutation is very recent and localized. Nonetheless, information about the virus-host association in the crAss-like family is scarce [12]. Thus, it is pivotal for the scientific community to put more effort in bacterial and viral isolation.

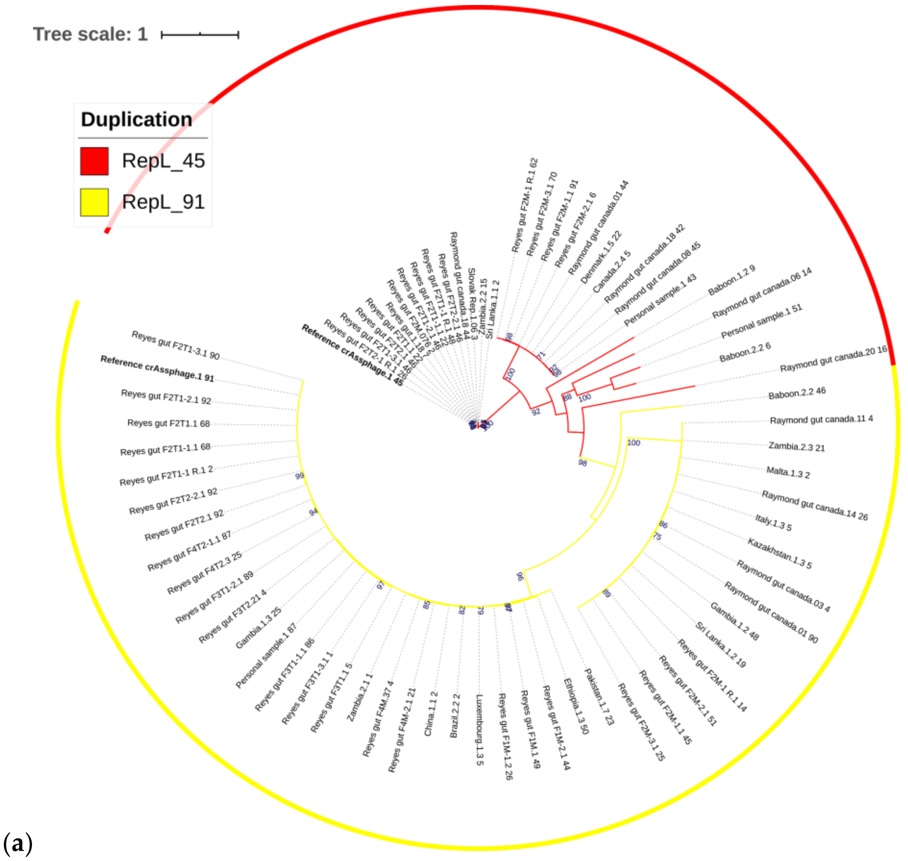
Tree scale: 1
Duplication
RepL_45
RepL_91

(**a**)

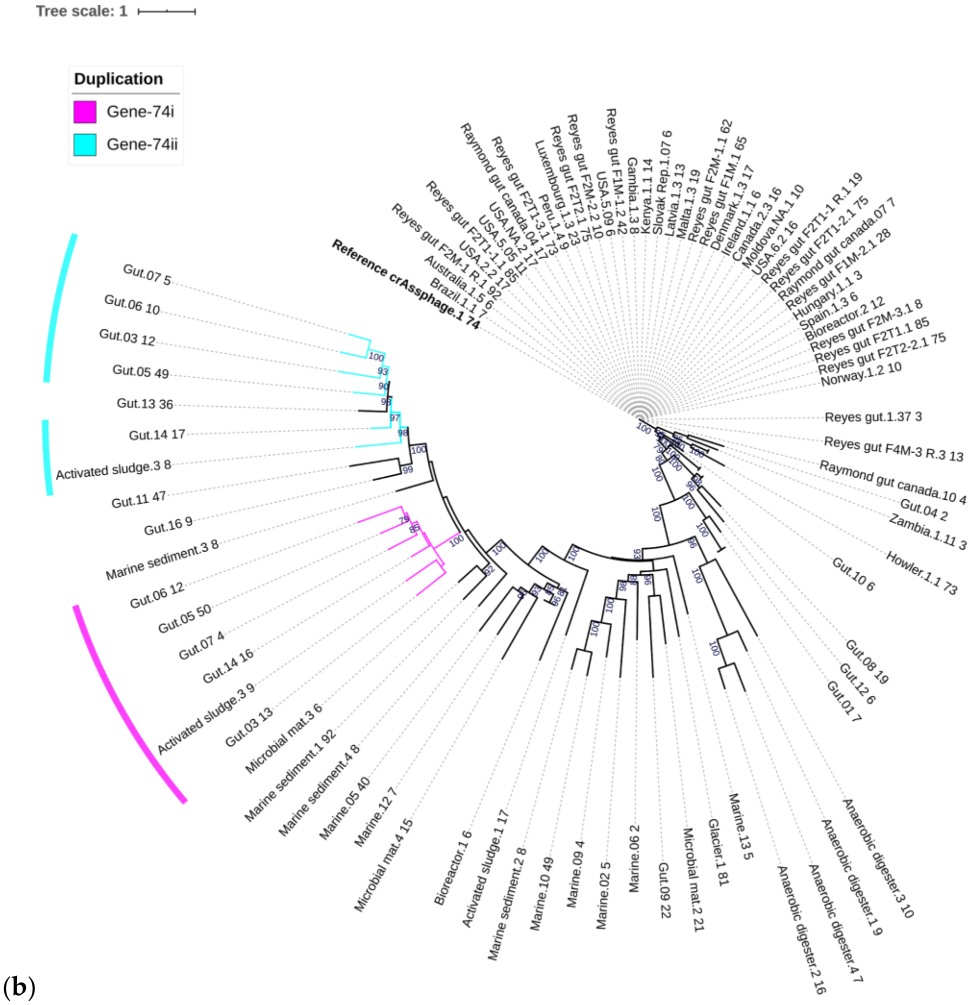
Tree scale: 1
Duplication
Gene-74i
Gene-74ii
Reference crAssphage.1 74
Gut.07 5
Gut.06 10
Gut.03 12
Gut.05 49
Gut.13 36
Gut.14 17
Activated sludge.3 8
Gut.11 47
Gut.16 9
Marine sediment.3 8
Gut.06 12
Gut.05 50
Gut.07 4
Gut.14 16
Activated sludge.3 9
Gut.03 13
Microbial mat.3 6
Marine sediment.1 92
Marine sediment.4 8
Marine.05 40
Marine.12 7
Microbial mat.4 15
Bioreactor.1 6
Activated sludge.1 17
Marine sediment.2 8
Marine.10 49
Marine.09 4
Marine.02 5
Marine.06 2
Gut.09 22
Microbial mat.2 21
Glacier.1 81
Marine.13 5
Anaerobic digester.2 16
Anaerobic digester.4 7
Anaerobic digester.1 9
Anaerobic digester.3 10
Gut.01 7
Gut.12 6
Gut.08 19
Gut.10 6
Howler.1.1 73
Zambia.1.11 3
Gut.04 2
Raymond gut canada.10 4
Reyes gut F4M-3 R.3 13
Reyes gut.1.37 3
Norway.1.2 10
Reyes gut F2T2-2.1 75
Reyes gut F2T1.1 85
Reyes gut F2M-3.1 8

(**b**)

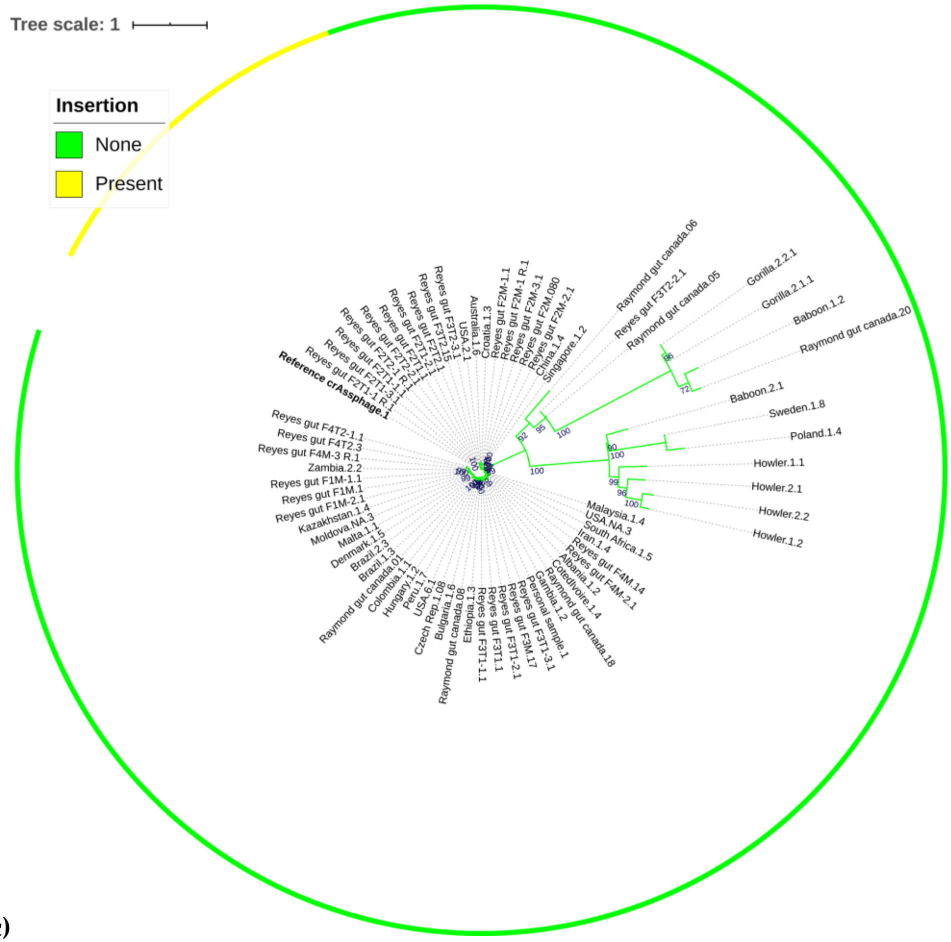

(**c**)

Figure S2. Unrooted phylogenetic trees of the genes that underwent gene-level evolutionary phenomena in crAss-like phages. The unrooted trees are displayed with the default root position. (**a**) Phylogenetic tree of the RepL genes. The shorter sequences, matching the Reference_crAssphage.1_45 ORF, are highlighted in red; the longer ones, which match both Reference_crAssphage.1_45 and Reference_crAssphage.1_91, are marked in yellow. (**b**) Part of the phylogenetic tree of Reference_crAssphage.1_74. Contigs coming from the same genome are highlighted in the same color. These genomes are very dissimilar from the reference crAssphage genome and, accordingly, the PSI-BLAST search provided significant matches only for capsid and structural proteins. (**c**) Phylogenetic tree of the Dut protein. The genomes in which the dut gene is intact are marked in green, whereas those in which the intron insertion has occurred are marked in yellow. They all come from genomes assembled from the metagenomes of a pair of twin sisters.

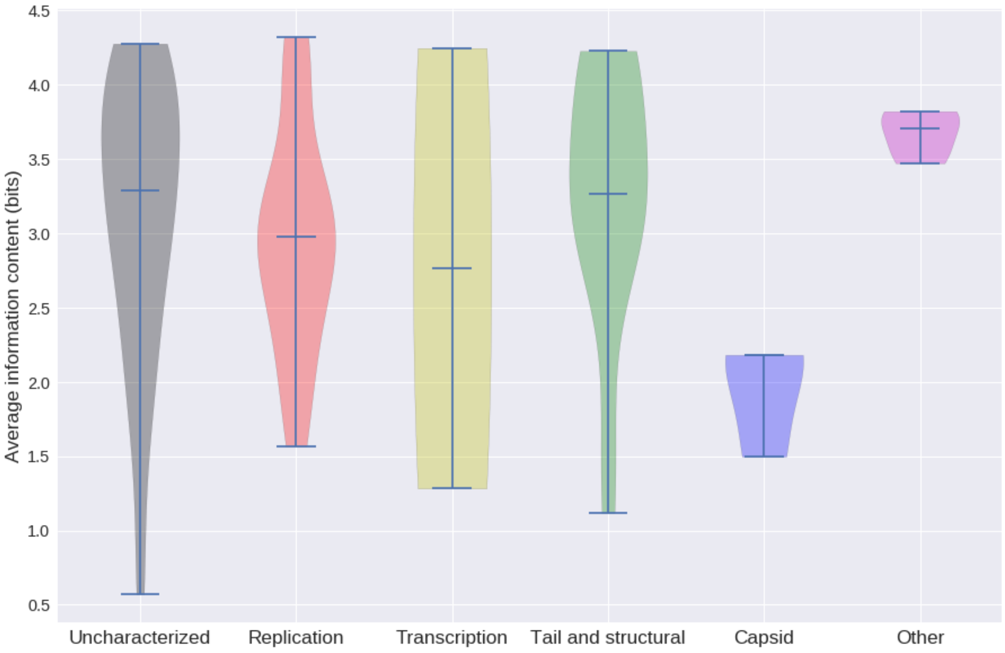

Figure S3. Violin plots showing the differences in distribution of Shannon information content across the different protein groups. The capsid proteins present an unexpectedly low degree of sequence conservation.

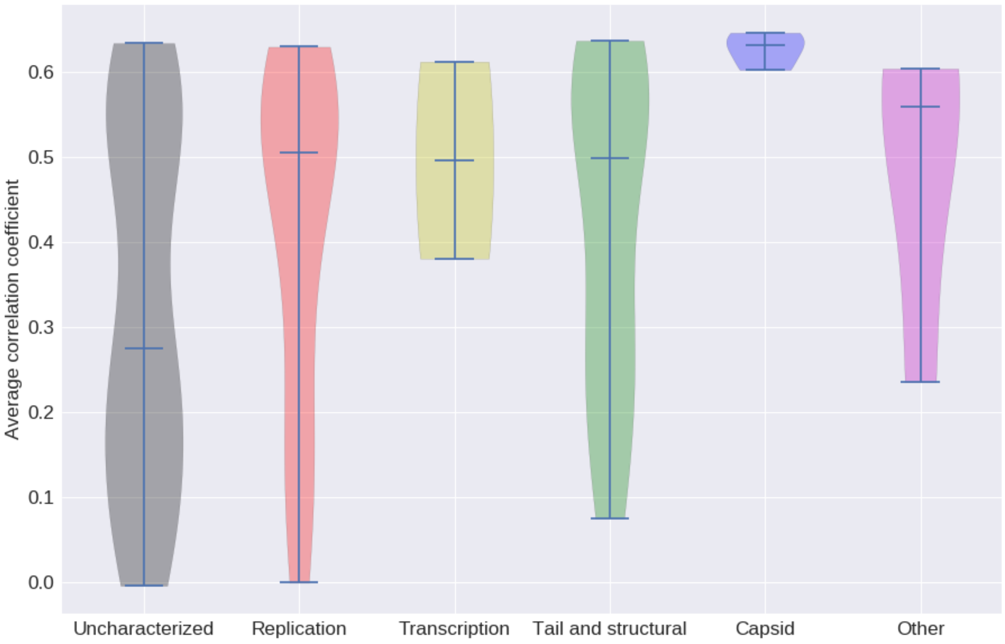

Figure S4. Violin plots showing the distribution of the average Mirrortree coefficient of each cluster of homologous proteins across the different functional categories, with the Capsid functional group sporting the narrowest distribution.

References

1. Kondrashov, F.A. Gene duplication as a mechanism of genomic adaptation to a changing environment. *Proc. R. Soc. B Biol. Sci.* **2012**, 279, 5048–5057, doi:10.1098/rspb.2012.1108.

2. Gao, Y.; Zhao, H.; Jin, Y.; Xu, X.; Han, G.-Z. Extent and evolution of gene duplication in DNA viruses. *Virus Res.* **2017**, 240, 161–165, doi:10.1016/j.virusres.2017.08.005.

3. Force, A.; Lynch, M.; Pickett, F.B.; Amores, A.; Yan, Y.L.; Postlethwait, J. Preservation of duplicate genes by complementary, degenerative mutations. *Genetics* **1999**, 151, 1531–1545.

4. Innan, H.; Kondrashov, F. The evolution of gene duplications: classifying and distinguishing between models. *Nat. Rev. Genet*. **2010**, 11, 97–108, doi:10.1038/nrg2689.

5. Roy, C.; Deo, I. Gene duplication: A major force in evolution and bio-diversity. *Int. J. Biodivers. Conserv*. **2014**, 6, 41–49, doi:10.5897/IJBC2012.090.

6. Corbett-Detig, R.B.; Hartl, D.L.; Sackton, T.B. Natural Selection Constrains Neutral Diversity across A Wide Range of Species. *PLOS Biol*. **2015**, 13, e1002112, doi:10.1371/journal.pbio.1002112.

7. Lanfear, R.; Kokko, H.; Eyre-Walker, A. Population size and the rate of evolution. *Trends Ecol. Evol*. **2014**, 29, 33–41, doi:10.1016/j.tree.2013.09.009.

8. Reyes, A.; Haynes, M.; Hanson, N.; Angly, F.E.; Heath, A.C.; Rohwer, F.; Gordon, J.I. Viruses in the faecal microbiota of monozygotic twins and their mothers. *Nature* **2010**, 466, 334–338, doi:10.1038/nature09199.

9. Edwards, R.A.; Vega, A.A.; Norman, H.M.; Ohaeri, M.; Levi, K.; Dinsdale, E.A.; Cinek, O.; Aziz, R.K.; McNair, K.; Barr, J.J.; et al. Global phylogeography and ancient evolution of the widespread human gut virus crAssphage. *Nat. Microbiol*. **2019**, 4, 1727–1736, doi:10.1038/s41564-019-0494-6.

10. Vértessy, B.G.; Tóth, J. Keeping Uracil Out of DNA: Physiological Role, Structure and Catalytic Mechanism of dUTPases. *Acc. Chem. Res.* **2009**, 42, 97–106, doi:10.1021/ar800114w.

11. Hizi, A.; Herzig, E. dUTPase: the frequently overlooked enzyme encoded by many retroviruses. *Retrovirology* **2015**, 12, 70, doi:10.1186/s12977-015-0198-9.

12. Koonin, E.V.; Yutin, N. The crAss-like Phage Group: How Metagenomics Reshaped the Human Virome. *Trends Microbiol*. **2020**, 28, 349–359, doi:10.1016/j.tim.2020.01.010.
